# Cost-effectiveness of switching from trivalent to quadrivalent inactivated influenza vaccines for the at-risk population in Italy

**DOI:** 10.1080/21645515.2018.1469368

**Published:** 2018-06-18

**Authors:** Francesco Saverio Mennini, Chiara Bini, Andrea Marcellusi, Alessandro Rinaldi, Elisabetta Franco

**Affiliations:** a Centre for Economics and International Studies-Economic Evaluation and Health Technology Assessment, University of Rome, Rome, Italy; b Institute for Leadership and Management in Health, Kingston University London, London, UK; c National Research Council, Institute for Research on Population and Social Policies, Rome, Italy; d Value & Access Country Lead, Vaccines, Sanofi s.p.a., Rome, Italy; e Department of Biomedicine and Prevention, Faculty of Medicine and Surgery, University of Rome, Rome, Italy

**Keywords:** cost-benefit analysis, chronic disease, elderly, Italy, influenza B, quadrivalent influenza vaccine, quality-adjusted life years

## Abstract

Seasonal influenza is caused by two subtypes of influenza A and two lineages of influenza B. Although trivalent influenza vaccines (TIVs) contain both circulating A strains, they contain only a single B-lineage strain. This can lead to mismatches between the vaccine and predominant circulating B lineages, a concern especially for at-risk populations. Quadrivalent influenza vaccines (QIVs) containing a strain from both B lineages have been developed to improve protection against influenza. Here, we used a cost-utility model to examine whether switching from TIV to QIV would be cost-effective for the at-risk population in Italy. Costs were estimated from the payer and societal perspectives. The discount rate for outcomes was 3.0%. Univariate and probabilistic sensitivity analyses were performed to examine the effects of variations in parameters. Switching from TIV to QIV in Italy was estimated to increase quality-adjusted life-years (QALYs) and produce cost savings, including €1.6 million for hospitalization and approximately €2 million in productivity. The incremental cost-effectiveness ratio was €23,426 per QALY from a payer perspective and €21,096 per QALY from a societal perspective. Switching to QIV was most cost-effective for individuals ≥ 65 years of age (€19,170 per QALY). Probabilistic sensitivity analysis showed that the switching from TIV to QIV would be cost-effective for > 91% of simulation at a maximum willingness-to-pay threshold of €40,000 per QALY gained. Although the model did not take herd protection into account, it predicted that the switch from TIV to QIV would be cost-effective for the at-risk population in Italy.

## Introduction

Vaccination is the most effective method to prevent influenza and its complications.^1^ In Italy, similar to other countries, the Ministry of Health recommend influenza vaccination for persons at risk for influenza complications due to underlying medical conditions, adults ≥ 65 years, pregnant women in the second or third trimester during the influenza season, persons with immunosuppression, residents of nursing homes and other long-term care facilities, and healthcare workers and other caregivers in contact with influenza patients.^2^ They also recommend influenza vaccination for all obese people (body mass index ≥ 30), government officials (e.g. police officers) working directly with the public, children and adolescents receiving long-term aspirin therapy who might be at risk for Reye syndrome, and people in contact with animals that may carry non-influenza viruses.

Until recently, influenza vaccines have been trivalent, containing two strains of influenza A and a single B strain lineage. However, since the early 2000s, two distinct genetic lineages of influenza B virus, Victoria and Yamagata, have co-circulated worldwide.^3^ This has complicated selection of the correct B lineage to include in the influenza vaccine and has resulted in frequent mismatches between the vaccine and the predominant circulating B strain.^4-9^

Quadrivalent influenza vaccines (QIVs) including both B strain lineages have been developed to avoid these B lineage mismatches.^3^ In Italy, a health technology assessment using a Markov model predicted that, based on a 100-year horizon for the full Italian population followed for a lifetime, switching from TIV to QIV would avoid 1,413,887 influenza cases, 169,638 cases with complications, and 20,905 influenza-related deaths. Furthermore, switching from TIV to QIV would be cost effective from the Italian National Health Service perspective at an incremental cost-effectiveness ratio (ICER) of €18,883 per quality-adjusted life year (QALY).^10^ For the 2015–2016 season, which had a 49% mismatch between the vaccine and circulating influenza B lineages, switching 9% of the vaccines used to QIV would have yielded a net savings of €674,089.^11^ A second assessment using a decision-tree model predicted that over the 10 influenza seasons between 2002–2003 and 2012–2013, had QIV been used instead of TIV in Italy, 231,133 influenza cases, 75,640 general practitioner (GP) consultations, 95,820 lost workdays, 5,344 hospitalizations, and 1,550 deaths would have been avoided, saving €1.6 million in GP costs, €16.3 million in hospitalization costs, and €21 million in lost productivity.^12^

These health analyses have estimated the impact of switching from TIV to QIV for the full Italian population, but its impact on the population targeted for influenza vaccination has not been described. In this study, we therefore used a cost-utility model to predict the public health impact and cost-effectiveness of switching from TIV to QIV for only the targeted population in Italy.

## Results

### Base case analysis

#### Health outcomes

For an average influenza season, the cost-utility model predicted that switching from TIV to QIV for the at-risk population in Italy would prevent an additional 2,401 cases of influenza not receiving medical consultation, 3,469 cases leading to a GP visit, 82 emergency department (ED) visits, 446 hospitalisations, and 133 deaths. This would also avoid 16,564 lost workdays (Table 1). The model also predicted that switching to QIV would lead to gains of 862 QALYs. Most of the improved health outcomes were in individuals aged ≥ 65 years.

**Table 1.** Health impact of switching from TIV to QIV for the targeted population in Italy during an average influenza season.

Age group	Non-consulting cases avoided	GP visits avoided	ED visits avoided	Hospitalizations avoided	Deaths avoided	Life years gained	QALYs gained	Work days saved
6 mo–4 y	20.5	29.9	0.3	0.5	0.0	0.0	0.4	—
5–19 y	75.5	112.0	0.5	0.7	0.0	0.2	1.8	720
20–49 y	306.8	454.9	2.2	3.2	0.6	14.6	21.4	6,734
50–64 y	414.8	615.3	2.2	18.4	2.7	50.2	55.4	9,110
≥ 65 y	1,583.5	2,256.9	77.2	423.7	129.8	819.5	783.0	—
Total	2,401	3,469	82	446	133	884	862	16,564

Abbreviations: ED, emergency department; GP, general practitioner; QALY, quality-adjusted life year; QIV, quadrivalent influenza vaccine; TIV, trivalent influenza vaccine

#### Cost utility

Switching from TIV to QIV would reduce productivity losses due to influenza by €2 million and save €1.6 million currently spent on hospitalisations (Table 2). The ICER was €23,426 per QALY gained from the payer perspective and €21,096 per QALY gained from the societal perspective. The ICER from a societal or payer perspective was €19,170 for adults aged ≥ 65 years. This is lower than the typical national threshold value of €30,000 per QALY gained.^13^

**Table 2.** Costs saved by switching from TIV to QIV for the targeted population in Italy during an average influenza season and incremental cost-effectiveness ratios.

	Costs saved					ICER (cost per QALY)	
Age group	GP visits	ED visits	Hospitalisations	Medication	Lost productivity due to influenza	Payer perspective	Societal perspective
6 mo–4 y	€ 619	€ 69	€ 1,862	€ 444	€ 0	€ 110,083	€ 110,083
5–19 y	€ 2,314	€ 116	€ 2,593	€ 1,635	€ 87,252	€ 148,021	€ 99,295
20–49 y	€ 9,398	€ 523	€ 11,904	€ 6,647	€ 816,617	€ 95,564	€ 57,315
50–64 y	€ 12,713	€ 519	€ 67,986	€ 8,960	€ 1,104,659	€ 51,067	€ 31,126
≥ 65 y	€ 46,628	€ 18,612	€ 1,567,598	€ 35,873	€ 0	€ 19,170	€ 19,170
Total	€ 71,671	€ 19,839	€ 1,651,944	€ 53,559	€ 2,008,527	€ 23,426	€ 21,096

Abbreviations: ED, emergency department; GP, general practitioner; ICER, incremental cost-effectiveness ratio; QALY, quality adjusted life year; QIV, quadrivalent influenza vaccine; TIV, trivalent influenza vaccine

### Sensitivity analyses

**Figure 1.**
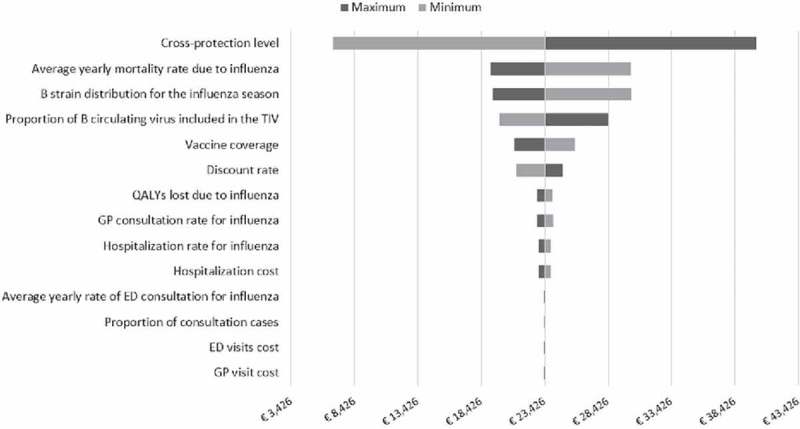
Deterministic sensitivity analysis (payer perspective). Abbreviations: ED, emergency department; GP, general practitioner; QALY, quality-adjusted life year; TIV, trivalent influenza vaccine.

**Figure 2.**
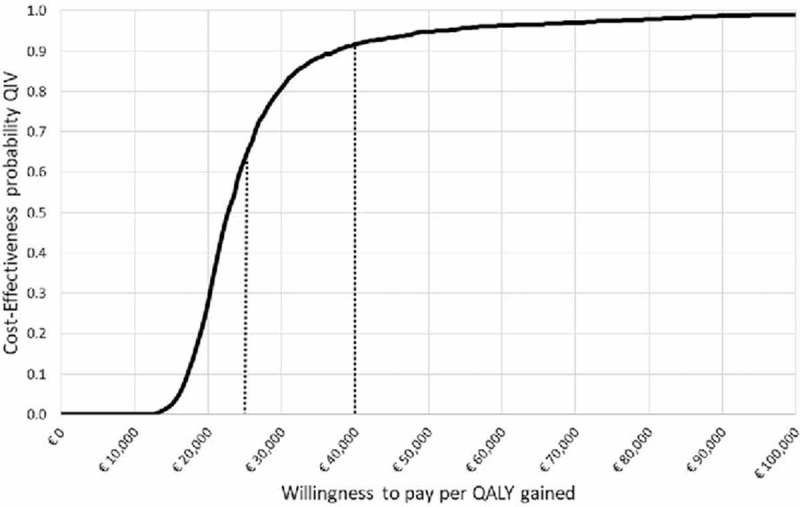
Cost-effectiveness acceptability curve (payer perspective). Abbreviations: QALY, quality-adjusted life year; QIV, quadrivalent influenza vaccination.

One-way deterministic analysis, conducted from the payer perspective, showed that the ICER is most sensitive to variation in the level of B strain cross-protection (from €6,689 in the low-case scenario to €40,087 in the high-case scenario), average annual mortality rate due to influenza (from €19,137 in the low-case scenario to €30,194 in the high-case scenario), and average level of mismatch (from €19,329 in the low-case scenario to €30,255 in the high-case scenario) (Fig. 1). Probabilistic sensitivity analysis confirmed that the switching from TIV to QIV would be cost-effective for > 63% of simulations at a minimum willingness-to-pay threshold of €25,000 per QALY gained and for > 91% of simulation at a maximum willingness-to-pay threshold of €40,000 per QALY gained (Fig. 2).

## Discussion

Our cost-utility model showed that switching from TIV to QIV for the targeted population in Italy is cost-effective. This would reduce productivity losses by €2 million and would save over €1.6 million currently spent on influenza-related hospitalisations. The ICER estimates (€23,426 per QALY gained from the payer perspective and €21,069 per QALY gained from the societal perspective) were below the typical national threshold values (€30,000 per QALY gained).^13^

In the UK, switching from TIV to QIV has been predicted to avoid 1,413,392 influenza cases, 41,780 hospitalizations, and 19,906 deaths over the lifetime horizon and to be cost-effective.^14^ In our study, switching from TIV to QIV was most beneficial to and most cost-effective for individuals ≥ 65 years of age, even though vaccine efficacy is reduced in this population.^15^

In contrast to a pervious analysis in the US by Reed et al.,^16^ we stratified by age group to account for differences in epidemiology, cross-protection, and vaccine coverage. However, our model does have several potential limitations. First, the estimates of vaccine coverage for most age groups (but not the ≥ 65 years group) had to be adjusted to fit the age groups used in the model. Although this may have distorted outcome estimates, the effect should be small for the base case, as indicated by the sensitivity analyses. Second, because of insufficient national data, we assumed that the estimated QALYs lost due to influenza would be the same as in a previous UK health technology assessment.^17^ Despite this limitation, the one-way sensitivity analysis in our model showed that this parameter did not result in significant variation in the ICER. Furthermore, due to limited data available, we used the average ED visit rate estimated from the available influenza seasons. However, also in this case, the one-way sensitivity analysis demonstrated the limited impact of this parameter in the ICER result. Finally, we assumed that the influenza vaccine effectiveness (IVE) was the same against influenza A(H1N1) and A(H3N2), but this should not have affected the comparison between TIV and QIV.

To study the effect of switching from TIV to QIV, we developed a model using a static approach, although a dynamic approach has been used to develop some models. Static models do not account for a herd effect, which can occur especially in paediatric populations, but this should have little effect on our results because the main target in this study was an elderly population. In any case, static models provide more conservative results than dynamic models.

In conclusion, switching to QIV in Italy's vaccination programme should provide health benefits to the targeted population and should also be cost effective for both healthcare providers and society. These benefits may also improve confidence in influenza vaccination in Italy and therefore vaccination coverage rates, which currently remain far below the Italian,^18^ European,^19^ and global^20^ targets of 75% for at-risk groups.

## Materials and methods

### Model structure

The objective of this study was to estimate the ICER of switching from TIV to QIV for the at-risk population in Italy. We developed a cost-utility model^21^ to calculate the health and economic impact of seasonal vaccination for an average seasonal influenza rate based on the observed epidemiology between 2003 and 2013 (excluding the 2009–2010 pandemic season). The cost-utility model was developed based on previous models designed to estimate the cost-effectiveness of QIV in the US,^16^ and Canada.^22^ It estimated health-related benefits by calculating avoided influenza-related cases, GP consultations, hospitalisations, deaths, and ED visits. Outputs also included the numbers of gained QALYs, gained life-years, and saved workdays. The cost-utility of QIV vs. TIV was calculated from both payer and societal perspectives. The population was stratified into age groups of 6 months–4 years, 5–19 years, 20–49 years, 50–64 years, and ≥ 65 years. The model included the 2016 Italian population susceptible to influenza complications and benefitting from vaccine reimbursement by the National Health Service.

### Model inputs and assumptions

Data from Italy were preferred, but when unavailable, data from other countries were used. Official Italian sources included the Italian Health Ministry (http://www.salute.gov.it/), the Italian Statistical Institute (www.istat.it/), the InfluNet Italian influenza surveillance network (http://www.iss.it/iflu/), and the Italian National Centre of Surveillance, Epidemiology, and Health Promotion (http://www.iss.it/).

### Population and life expectancy

The size of the at-risk population in Italy was based on population statistics for 2016,^23^ combined with the proportion considered at risk each year^24^ (Table 3). Individuals were considered at risk for influenza complications if they were ≥ 65 years of age or had an underlying chronic disease as specified by the Italian Ministry of Health.^18^ Life expectancy data for Italy were obtained from national statistics.^25^

**Table 3.** Input values.

			PSA^a^	
Model input	Baseline value	DSA range	Distribution type	Parameters
At-risk population in Italy, n				
6 mo–4 y	213,555	—	—	—
5—19 y	1,000,788	—	—	—
20—49 y	4,449,039	—	—	—
50—64 y	6,277,505	—	—	—
≥ 65 y	13,369,754	—	—	—
Life expectancy, y				
6 mo—4 y	80.46	—	—	—
5—19 y	70.63	—	—	—
20—49 y	47.02	—	—	—
50—64 y	27.73	—	—	—
≥ 65 y	12.59	—	—	—
Average yearly influenza-related GP visits for influenza per 100,000 individuals, n				
6 mo—4 y	5512.5	±25%	Normal+ (µ,σ)	(1.00;0.05)
5—19 y	3975	±25%	Normal+ (µ,σ)	(1.00;0.05)
20—49 y	1725	±25%	Normal+ (µ,σ)	(1.00;0.05)
50—64 y	1725	±25%	Normal+ (µ,σ)	(1.00;0.05)
≥ 65 y	950	±25%	Normal+ (µ,σ)	(1.00;0.05)
Average yearly influenza-related ED visits per 100,000 individuals, n				
6 mo—4 y	48.1	±25%	Normal+ (µ,σ)	(1.00;0.05)
5—19 y	15.2	±25%	Normal+ (µ,σ)	(1.00;0.05)
20—49 y	7.5	±25%	Normal+ (µ,σ)	(1.00;0.05)
50—64 y	5.5	±25%	Normal+ (µ,σ)	(1.00;0.05)
≥ 65 y	30.4	±25%	Normal+ (µ,σ)	(1.00;0.05)
Average yearly influenza-related hospitalizations per 100,000 individuals, n				
6 mo—4 y	92.6	±25%	Normal+ (µ,σ)	(1.00;0.05)
5—19 y	24.9	±25%	Normal+ (µ,σ)	(1.00;0.05)
20—49 y	12.3	±25%	Normal+ (µ,σ)	(1.00;0.05)
50—64 y	52	±25%	Normal+ (µ,σ)	(1.00;0.05)
≥ 65 y	178.7	±25%	Normal+ (µ,σ)	(1.00;0.05)
Average yearly influenza-related deaths per 100,000 individuals, n				
6 mo—4 y	0	±25%	Normal+ (µ,σ)	(1.00;0.05)
5—19 y	0.2	±25%	Normal+ (µ,σ)	(1.00;0.05)
20—49 y	2.1	±25%	Normal+ (µ,σ)	(1.00;0.05)
50—64 y	7.3	±25%	Normal+ (µ,σ)	(1.00;0.05)
≥ 65 y	55	±25%	Normal+ (µ,σ)	(1.00;0.05)
Utility for at-risk population				
6 mo—4 y	0.95	—	Beta (α,β)	(−0.1543;−0.0081)
5—19 y	0.95	—	Beta (α,β)	(−0.1548;−0.0081)
20—49 y	0.942	—	Beta (α,β)	(−0.0059;−0.0004)
50—64 y	0.913	—	Beta (α,β)	(0.4791;0.0457)
≥ 65 y	0.872	—	Beta (α,β)	(1.1752;0.1724)
QALYs lost due to influenza, y				
6 mo—4 y	0.0146	0.0110–0.0183	Beta (α,β)	(15.75;1063.13)
5—19 y	0.0146	0.0110–0.0183	Beta (α,β)	(15.75;1063.13)
20—49 y	0.0174	0.0131–0.0218	Beta (α,β)	(15.70;886.83)
50—64 y	0.0174	0.0131–0.0218	Beta (α,β)	(15.70;886.83)
≥ 65 y	0.0293	0.0220–0.0366	Beta (α,β)	(15.50;513.57)
Influenza vaccination coverage, %				
6 mo—4 y	9.66	7.25–12.08	Beta (α,β)	(14.35;134.27)
5—19 y	10.86	8.15–13.58	Beta (α,β)	(14.15;116.17)
20—49 y	18.60	13.95–23.25	Beta (α,β)	(12.83;56.18)
50—64 y	18.60	13.95–23.25	Beta (α,β)	(12.83;56.18)
≥ 65 y	49.90	37.43–62.38	Beta (α,β)	(7.51;7.54)
Proportion of influenza cases requiring medical consultation, %				
6 mo—4 y	59.58	44.69–74.48	Beta (α,β)	(5.8714;3.9832)
5—19 y	59.82	44.87–74.78	Beta (α,β)	(5.8306;3.9163)
20—49 y	59.84	44.88–74.80	Beta (α,β)	(5.8275;3.9113)
50—64 y	59.82	44.87–74.78	Beta (α,β)	(5.8306;3.9163)
≥ 65 y	59.58	44.69–74.48	Beta (α,β)	(5.8714;3.9832)
Mean daily per-person productivity (€)				
6 mo—4 y	0.00	—	—	—
5—19 y	121.26	—	—	—
20—49 y	121.26	—	—	—
50—64 y	121.26	—	—	—
≥ 65 y	0.00	—	—	—
Cost of resources used (€)				
GP visits	20.66	15.50–25.83	Gamma (µ,σ)	(20.66;2.64)
ED visits	241.00	180.75–301.25	Gamma (µ,σ)	(241.00;30.74)
Hospitalization	3.700.00	2775.00–4625.00	Gamma (µ,σ)	(3700.00;471.94)
Vaccine cost (€)				
TIV	5.39^b^	—	—	—
QIV	11.08^b^	—	—	—
Medication costs (€)				
GP consultation	12.40	—	Gamma (µ,σ)	(12.40;1.58)
ED consultation	40.74	—	Gamma (µ,σ)	(40.74;5.20)
No consultation	3.00	—	Gamma (µ,σ)	(3.00;0.38)
Lost workdays due to medical consultation for influenza				
6 mo—4 y	0.00			
5—19 y	0.26	—	—	—
20—49 y	0.26	—	—	—
50—64 y	0.26	—	—	—
≥ 65 y	0.00			
Employment rate (%)				
6 mo—4 y	0.0	—	—	—
5—19 y	15.6	—	—	—
20—49 y	60.5	—	—	—
50—64 y	48.2	—	—	—
≥ 65 y	0.0	—	—	—
Working hours per week				
6 mo—4 y	0.0	—	—	—
5—19 y	36.0	—	—	—
20—49 y	36.0	—	—	—
50—64 y	36.0	—	—	—
≥ 65 y	0.0	—	—	—
Discount rate	0.03	0.00—0.05	—	—
Relative circulating level of B strain vs. total influenza, %				
2003—2004	1.00	0.75—1.25	Beta (α,β)	(15.83;1567.17)
2004—2005	16.50	12.38—20.63	Beta (α,β)	(13.19;66.77)
2005—2006	59.80	44.85—74.75	Beta (α,β)	(5.83;3.92)
2006—2007	2.10	1.58—2.63	Beta (α,β)	(15.64;729.26)
2007—2008	38.60	28.95—48.25	Beta (α,β)	(9.43;15.01)
2008—2009	16.90	12.68—21.13	Beta (α,β)	(13.12;64.54)
2010—2011	1.30	0.98—1.63	Beta (α,β)	(15.77;1197.99)
2011—2012	28.80	21.60—36.00	Beta (α,β)	(11.10;27.45)
2012—2013	38.80	29.10—48.50	Beta (α,β)	(9.40;14.83)

Abbreviations: DSA, deterministic sensitivity analysis; ED, emergency department; GP, general practitioner; Normal+, normal positive; PSA, probabilistic sensitivity analysis; QALY, quality-adjusted life year; SD, standard deviation.

a For positive normal distribution, simulation values < 0 were assigned a value of 0. Beta distributions show their (α,β) parameterisation, where α = number of success, β = number of failures.

b This price corresponds to the ex-factory price per dose negotiated by the Italian Agency for Medicines. In Italy, the vaccination programme is financed at the regional level, and local health agencies obtain the vaccine at a different price for each region. For this analysis, prices were as reported in a recent Italian analysis.^11^

### Influenza-related health parameters

Average yearly rates of GP consultations were from the InfluNet surveillance system of the Istituto Superiore di Sanità.^26^ Age groups, hospitalizations, and deaths due to influenza in Italy were derived from Uhart et al.^12^ (Table 4). The estimated mortality rate was assumed to be zero for children ≤ 4 years of age. The rate of influenza-related ED visits in Italy was from Epicentro, the epidemiology portal for the Istituto Superiore di Sanità.^27^ Because the weekly bulletins were only available for the 2012–2013, 2013–2014, and 2014–2015 influenza seasons, the rate was calculated as the mean of the three available influenza seasons and was assumed consistent for all the seasons analysed.

**Table 4.** Influenza vaccine effectiveness for trivalent influenza vaccine in at-risk individuals for A strains and matched and mismatched B lineage strains.

Age group	A(H1N1)^a^	A(H3N2)^a^	Matched B^b^	Mismatched B^b^
6 mo – 4 y	59.0%	59.0%	66.0%	44.0%
5 – 19 y	59.0%	59.0%	66.0%	44.0%
20 – 49 y	61.0%	61.0%	77.0%	52.0%
50 – 64 y	61.0%	61.0%	73.0%	49.0%
≥ 65 y	58.0%	58.0%	66.0%	44.0%

a From Uhart et al.^12^ Influenza vaccine effectiveness was assumed to be the same for each A strain.

b From Tricco et al.^33^

Utility rates for influenza were based on estimates of the adult population in Italy using EQ-5D-5L.^28^ QALYs lost due to influenza were based on data from a cost-utility analysis for Ontario.^29^

Influenza vaccination coverage rates were from the previous budget impact analysis of QIV in Italy^11^ for at-risk individuals 6 months to 19 years of age; from the National Centre of Surveillance, Epidemiology, and Health Promotion for at-risk individuals 20 to 49 and 50 to 64 years of age^30^; and from the Italian Ministry of Health for individuals ≥ 65 years of age.^31^

Proportions of influenza cases resulting in medical consultation were from a previous cost-effectiveness analysis of influenza vaccination in Italy^10^ and a 2001 study on the epidemiology, natural history, and resource use associated with influenza in the general population setting in Italy.^32^

The proportion of influenza illness due to influenza A, A(H1N1), A(H3N2), B, B/Yamagata, and B/Victoria in Italy for each year between 2003–2004 and 2012–2013 was from Uhart et al.^12^ and InfluNet^26^ (Table S1). The level of match between the TIV and circulating B strain lineages in Italy for each season was from Barbieri et al.^10^

### IVE

The average IVE for TIV and QIV for each age group against influenza-related GP visits, ED visits, hospitalization, and death (Table S2) were calculated following the method used for the Canadian model^22^ and using the IVE in each age group against influenza A from Uhart et al.^12^ and IVE against matched and mismatched B-lineage influenza from a systematic review by Tricco et al.^33^ (Table 4). IVE was assumed to be the same against influenza A(H1N1) and A(H3N2). IVE against each outcome in each age group was calculated as described for the Canadian^22^ and US^16^ models.

### Costs

#### Direct costs

Vaccine costs are highly variable in Italy due to regional competitive price fixing. For this analysis, prices reported in a recent Italian analysis^10^ were used. Medication costs were set as described in the Pitrelli et al.^11^ (Table 3). Costs of GP visits^34^ and ED visits^35^ were based on data from the Ministry of Health (Table 3). The cost of hospitalization for influenza was based on an Italian health technology assessment^36^ and on costs specified by the Ministry of Health (Table 3).^36^

#### Indirect costs

The costs of productivity loss were calculated from the estimated number of workdays lost in Italy from October 2014 to January 2015 (1.5 million),^37^ the estimated average daily productivity costs for Italy in 2016 (€121.26),^38^ and assuming 36 working hours per week. Children ≤ 4 years of age and adults ≥ 65 years of age were assumed to not be working.

### Analyses

#### Base case

Cost-utility analysis for the base case was carried out from the National Health Service (payer) and societal perspectives. For the payer perspective, the model only included estimated health costs directly associated with treating, managing, and caring for patients with influenza. For the societal perspective, the model included indirect costs, specifically, loss of productivity due to influenza among the employed population. The effects were discounted by a 3.0% annual rate.^13^^,^^21^ The analysis included patients vaccinated with TIV and QIV as two separate cohorts considering the seasonal effects of the influenza vaccination and that patients would benefit from only a single vaccination each season.

#### Sensitivity analyses

Key model input parameters were varied individually in deterministic sensitivity analyses to measure their influence on the model. A variation of ± 25% was assumed for all parameters except for discount rate, for which the value was varied from 0% to 5%, and TIV IVE against a mismatched type B lineage (i.e. cross-protection), for which the value was varied from 0% to 80% of the matched type B value.

To assess uncertainty in parameters, probabilistic sensitivity analysis was conducted varying all the parameters together, each according to a defined probabilistic distribution. Two thousand Monte Carlo simulations were performed to generate the cost-effectiveness acceptability curve. The probabilistic results for cost-effectiveness are reported for both the minimum and maximum acceptability thresholds according to the Associazione Italiana di Economia Sanitaria (Italian Health Economics Association) guidelines.^13^ Based on the thresholds described by the UK National Institute of Health and Care Excellence,^39^ the minimum threshold is €25,000 and the maximum is €40,000 per QALY gained.
